# Microbe-Host Communication by Small RNAs in Extracellular Vesicles: Vehicles for Transkingdom RNA Transportation

**DOI:** 10.3390/ijms20061487

**Published:** 2019-03-25

**Authors:** Heon-Jin Lee

**Affiliations:** 1 Department of Microbiology and Immunology, School of Dentistry, Kyungpook National University, Daegu 41940, Korea; heonlee@knu.ac.kr; Tel.: +82-53-660-6832; 2 Brain Science and Engineering Institute, Kyungpook National University, Daegu 41940, Korea

**Keywords:** microRNA (miRNA), outer membrane vesicle (OMV), small RNA (sRNA), extracellular RNA (exRNA), extracellular vesicle (EV)

## Abstract

Extracellular vesicles (EVs) are evolutionary well-conserved nano-sized membranous vesicles that are secreted by both prokaryotic and eukaryotic cells. Recently, they have gained great attention for their proposed roles in cell-to-cell communication, and as biomarkers for human disease. In particular, small RNAs (sRNAs) contained within EVs have been considered as candidate interspecies-communication molecules, due to their demonstrated capacity to modulate gene expression in multiple cell types and species. While research into this field is in its infancy, elucidating the mechanisms that underlie host–microbe interactions and communications promises to impact many fields of biological research, including human health and medicine. Thus, this review discussed the results of recent studies that have examined the ways in which EVs and sRNAs mediate ‘microbe–host’ and ‘host–microbe’ interspecies communication.

## 1. Introduction

Extracellular RNAs (exRNAs) encompass secreted RNAs that are thought to be mainly encased within extracellular vesicles (EVs) or are otherwise tightly bound to cellular proteins and lipids [1]. EVs are a heterogeneous population of nano-sized membranous vesicles of various types, including eukaryotic cell exosomes, microvesicles, and gram-negative bacterial outer-membrane vesicles (OMVs) [2]. The majority of EV RNA content comprises small RNAs (sRNAs), such as microRNAs (miRNAs), and miRNA-sized sRNAs (msRNAs) [3,4]. These have recently gained attention due to their proposed gene-regulatory roles [2]; for example, miRNAs contained in EVs have been recently suggested as biomarkers for cancer and other diseases [5,6]. miRNAs are initially processed from precursor miRNAs (pre-miRNAs), before being processed to their final mature form by the RNase-III enzyme, Dicer [7,8]. Once mature, they are typically 18–24 nt in length, which is similar to the size of siRNAs, as well as that of most polymerase chain reaction (PCR) primers. The latter are designed to be long enough to achieve adequate specificity, but also short enough to bind easily to target sequences at standard annealing temperatures [9,10]. It may be that the common lengths of miRNAs have functionally evolved as the minimum size required to facilitate target-gene specificity, while avoiding cellular immune responses.

Exosomes have lipoprotein envelopes that are similar to cell membranes and are derived from intracellular multivesicular bodies (MVBs). They carry and protect miRNAs from RNases in the extracellular environment [11,12]. miRNA effector complexes, including Argonaute 2 (Ago2), have been shown to be associated with MVBs and are thought to be required for miRNA loading and/or sorting [13,14]. Notably, OMV biogenesis is species-specific, in that it varies with the structure and characteristics of bacterial cell-wall components, including the peptidoglycan layer and lipopolysaccharides (LPS) [15]. Microvesicles are shed from the surface of plasma membranes of many cell types and are generally larger (~1000 nm) than other types of EVs [16]. In contrast to exosomes, microvesicles bud directly from the plasma membrane, and thus, usually leave membrane proteins intact [17]. Once shed, microvesicles can transport genetic material, including miRNAs and cytosolic and membrane proteins, such as integrins, MHC (Major histocompatibility complex) class-I molecules, and soluble proteins [18]. However, given that current techniques are limited in their ability to discriminate between different EV types, this review will refer to all such vesicles as ‘EVs’.

Many unexpected and intriguing types of sRNA-mediated interspecies communication have been demonstrated to date. For example, several papers have postulated that dietary miRNAs in cow milk and chicken eggs may regulate human gene expression [19]. Similarly, interactions exist between plant and fungi by sRNAs and RNA silencing machinery in broad hosts [20,21]. Moreover, a very recent paper described host–pathogen interactions by which miRNAs present in the saliva of anopheline mosquitoes may regulate host mRNAs involved in immune responses [22]. Finally, free exRNAs that are not contained within EVs have also been shown to associate with EV membranes [23,24], which suggests that microbial exRNAs may be incorporated into host EVs, or vice versa.

Of the many sRNA-mediated host–microbe interactions reported to date, this review focuses on the recently identified role of exRNAs in facilitating mutual communication between microbes and hosts.

## 2. EV Biogenesis, sRNA Sorting, and Host-Cell Entry

Perhaps due to their limited space and/or special selection mechanisms, transfer RNA (tRNA) fragments and miRNAs have been found to occur more frequently in exosomes than other sRNAs, such as messenger RNAs (mRNAs) and ribosomal RNAs (rRNAs) [25,26]. Exosomes are mainly produced from MVBs, and the process involves endosomal-sorting complexes that regulate the selection or ‘sorting’ of cellular miRNAs for secretion from the endosomal membrane compartment after fusion with MVBs. Notably, this sorting process often ensures that cellular and exosomal miRNA compositions are different [2,12,27]. To date, several proteins, including the synaptotagmin binding cytoplasmic RNA interacting protein (SYNCRIP) [28], major vault protein (MVP) [29], Kirsten rat sarcoma (KRAS) [30], and heterogeneous nuclear ribonucleoprotein A2/B1 (hnRNPA2B1) [31], have been shown to mediate miRNA sorting, and 3′ uridylation has been reported to be enriched amongst exosomal miRNAs [32]. A study has also suggested that particular miRNAs may be differentially sorted between the cytoplasm and exosomes according to the levels of mRNA target transcripts that are present in each location [33], in a manner similar to the target transcript-driven miRNA arm-selection mechanism that we previously described [34].

Conversely, bacterial EV biogenesis mechanisms have been suggested to be species-specific, and likely related to the physical structure the of the cell-wall peptidoglycan layer, and/or its LPS or membrane transporter-protein composition (see [15,35] for more details). This is because in gram-negative bacteria, the outer membrane must detach from beneath the peptidoglycan layer, form a vesicular shape, and then undergo fission to form an EV. Notably, however, gram-positive bacteria (which lack outer membranes) also produce EVs, suggesting that EV generation methods may be evolutionary conserved [36]. To date, neither the mechanisms underlying sRNA sorting into bacterial EVs nor relative bacterial-cell and EV sRNA profiles have been clarified.

It is well recognized that EVs can enter host cells via various endocytic routes, such as in a clathrin- or caveolin-mediated manner, via a lipid raft, or via membrane fusion (see details in [37,38]). Once inside target recipient (host) cells, EVs release their components, many of which (such as mRNAs and miRNAs) begin to function in the host cell, for example, being translated, and modulating the transcription of their target mRNAs in the host-cell, respectively [39] (Table 1).

## 3. Regulation of Host Genes by sRNAs Contained within Bacterial EVs

Typically, humans carry high levels of exogenous commensal bacteria-derived sRNAs in their biofluids (e.g., saliva and plasma) [45]. This finding is not surprising given that there are estimated to be up to 100 times more microbes than host cells in the human body [46].

Pathogen-derived RNAs are well established to induce innate immunity in host cells by activating Toll-like receptors (TLRs) and other cytosolic pattern recognition receptors (PRRs), such as retinoic acid-inducible gene-1 (RIG-1) [47,48]. For example, the murine TLR7 protein (human orthologue, TLR7/8) is predominantly responsive to viral single-stranded RNAs (ssRNAs), as well as to streptococcal bacterial RNAs in dendritic cells [47,49], supporting the hypothesis that microbial exRNAs activate host signal cascades via PRRs. Furthermore, TLR systems can be regulated by miRNAs [50], suggesting that RNA-PRR networks may be a common axis for bacteria–host communication.

Internalized viruses and bacteria have been demonstrated to express sRNAs that act as miRNAs. For example, many viruses encode miRNAs that are expressed in host cells, and these miRNAs facilitate viral replication and survival, and suppress or regulate host immunity [51,52]. A previous study showed that once internalized into host THP-1 macrophage cells, the bacteria, *Mycobacterium marinum*, expresses sRNAs that are bound by the host RNA-induced silencing complex (RISC). This observation supports the existence of bacterial miRNAs, which regulate (inhibit) host gene expression [44]. Similarly, sRNAs produced by another intracellular bacterial pathogen, *Salmonella enterica,* have been identified via dual RNA-seq. One of these, namely *PinT*, has been shown to both regulate the expression of host genes, and to mediate the activity of invasion-associated bacterial effectors and virulence genes required for intracellular survival [43].

Our group has also recently shown that periodontal pathogens produce miRNA-sized sRNAs (msRNAs) that can be secreted in OMVs, and thereby spontaneously transferred into eukaryotic cells. Moreover, we also demonstrated that the ectopic expression of highly expressed periodontal-pathogen msRNAs in T lymphocytes induced the production of cytokines, such as interleukin (IL)-5, IL-13, and IL-15 [41]. Similarly, another study showed that *Pseudomonas aeruginosa*-derived methionine tRNA can be transferred in OMVs into human epithelial airway cells, and thereby reduce their secretion of IL-8 [42]. Finally, an aggressive fungal pathogen, *Botrytis cinereal*, has been shown to harbor virulent sRNAs that bind the RISC to inhibit host-immunity genes [21]. Together, these results suggest that microbial msRNAs may act as communication molecules to mediate bacteria–host interactions. Similarly, animal parasites are also known to produce EV-contained circulating miRNAs and/or sRNAs in their hosts [53], some of which have been shown to modulate hosts’ innate immunity [54].

Thus, these findings oppose the traditional view on immune system regulation by revealing the mechanisms by which our body resists microbial attacks in an active manner.

## 4. Regulation of Bacterial Genes by sRNAs in Host EVs

Research exploring the mechanisms by which host miRNAs regulate bacterial gene expression has only recently begun. Traditionally, the lack of RNA interference (RNAi) machinery in bacteria led researchers to consider the possibility of host miRNA-mediated bacterial gene regulation skeptically. However, this changed when Liu et al. showed that fecal miRNAs secreted by gut epithelial cells can enter gut bacteria, and thereby regulate bacterial gene expression and growth [40], suggesting that bacteria may employ unique sRNA-driven transcriptional regulatory mechanisms. Supporting this hypothesis, Liu et al. also demonstrated that mice carrying miRNA-deficient gut endothelial cells exhibited uncontrolled gut microbiota growth and colitis, which was ameliorated upon transplantation of wild-type fecal miRNAs [40]. Gut microbiota have also been shown to influence host miRNA expression via TLR-dependent innate immunity pathways [55]. Additionally, miRNAs associated with several bacterial taxa have been shown to be differentially expressed in patients with colorectal cancer [56], suggesting that the abundance of gut microbes may be modulated by host miRNA-mediated communication. Given that all cells can produce and secrete miRNAs, such inter-species gene regulation by host miRNAs may not be limited to the gut microbiota. Indeed, the normal flora in the human body comprises an enormous number and range of bacteria, and we speculate that many of these may be affected by host miRNA. It is also possible that host-derived circulating miRNAs may indirectly influence the bacterial flora in ways that have not yet been elucidated.

## 5. Concluding Remarks

**Figure 1:**
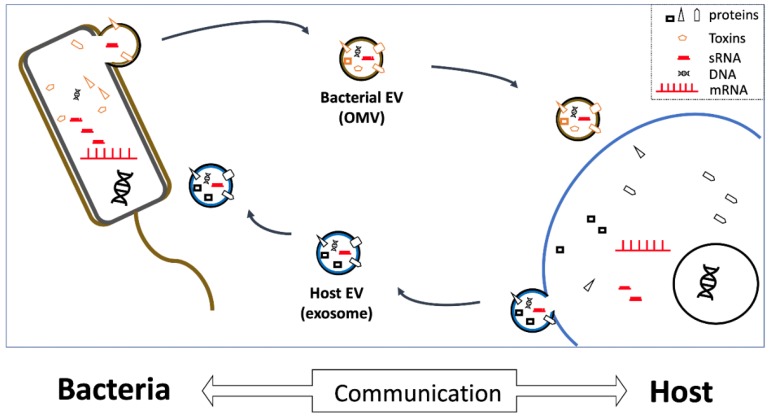
Vesicles as vehicles. Extracellular vesicles (EVs) secreted by both host and bacterial cells contain extracellular RNAs (exRNAs), as well as various other proteins and DNAs. EVs can be taken up by bacteria, or by host cells (in biofluids), and thereby likely critically mediate microbe-host communication by releasing small exRNAs capable of regulating recipient-cell gene expression. OMV, outer-membrane vesicle; sRNA, small RNA; mRNA, messenger RNA.

There are many possible avenues for interspecies communication between microbes and human cells; however, sRNAs represent particularly good candidates for a “common language” between species (Figure 1), because they are produced by every living organism, and can be transported throughout the human body by EVs. While traditional views of molecular biology have emphasized the importance of protein-coding and mRNAs for cellular and organismal function, recent studies demonstrate that understanding microbe–human interactions will likely be essential to elucidate the underlying mechanisms, and thus effectively treat human pathogenic diseases.

## Figures and Tables

**Table 1:** Examples of host–microbe interspecies communication by extracellular RNAs.

Communication Direction	Communication Function	Target	Reference
Host to microbes	Fecal miRNAs enter gut bacteria and regulate both gene transcript levels and bacterial growth	Gut	[40]
Microbes to host	Periodontal pathogens-derived sRNAs regulate T-cell cytokine production	Mouth	[41]
	*Pseudomonas aeruginosa*-derived sRNAs (in OMVs) reduce IL-8 production in human epithelial cells	Airway epithelial cells	[42]
	Host cell-internalized *Salmonella enterica* sRNA (namely ‘PinT’) regulate host-cell JAK-STAT signaling	HeLa cells	[43]
	Host cell-internalized *Mycobacterium marinum* sRNAs act like miRNAs, and bind to the host RISC	Macrophage-like cells (THP-1)	[44]
	Fungal sRNAs repress host (plant) innate immunity	*Arabidopsis* and tomato cells	[21]

sRNA, small RNA; T cells, thymus cells; OMV, outer membrane vesicle; IL, interleukin; JAK-STAT, Janus kinase/signal transducers and activators of transcription; HeLa, Henrietta Lacks cell line; miRNA, micro-RNAs; RISC, RNA-induced silencing complex; THP-1, human monocytic cell line.

## Abbreviations

EV	Extracellular vesicle
exRNA	Extracellular RNA
MHC	Major histocompatibility complex
miRNA	microRNA
msRNA	miRNA-sized small RNA
OMV	Outer membrane vesicle
PRR	Pattern recognition receptor
RISC	RNA-induced silencing complex
RNAi	RNA interference
sRNA	Small RNA
ssRNA	single-stranded RNA
TLR	Toll-like receptor

## References

[1] Patton J.G., Franklin J.L., Weaver A.M., Vickers K., Zhang B., Coffey R.J., Ansel K.M., Blelloch R., Goga A., Huang B. (2015). Biogenesis, delivery, and function of extracellular RNA. J. Extracell. Vesicles.

[2] Choi J.-W., Um J.-H., Cho J.-H., Lee H.-J. (2017). Tiny RNAs and their voyage via extracellular vesicles: Secretion of bacterial small RNA and eukaryotic microRNA. Exp. Biol. Med..

[3] Garcia-Contreras M., Shah S.H., Tamayo A., Robbins P.D., Golberg R.B., Mendez A.J., Ricordi C. (2017). Plasma-derived exosome characterization reveals a distinct microRNA signature in long duration Type 1 diabetes. Sci. Rep..

[4] Ghosal A., Upadhyaya B.B., Fritz J.V., Heintz-Buschart A., Desai M.S., Yusuf D., Huang D., Baumuratov A., Wang K., Galas D. (2015). The extracellular RNA complement of Escherichia coli. Microbiologyopen.

[5] Michael A., Bajracharya S.D., Yuen P.S.T., Zhou H., Star R.A., Illei G.G., Alevizos I. (2010). Exosomes from human saliva as a source of microRNA biomarkers. Oral. Dis..

[6] Xie Z., Chen G., Zhang X., Li D., Huang J., Yang C., Zhang P., Qin Y., Duan Y., Gong B., Li Z. (2013). Salivary microRNAs as promising biomarkers for detection of esophageal cancer. PLoS ONE.

[7] Bartel D.P. (2004). MicroRNAs: Genomics, biogenesis, mechanism, and function. Cell.

[8] Lee H.-J. (2013). Exceptional stories of microRNAs. Exp. Biol. Med..

[9] Dieffenbach C.W., Lowe T.M., Dveksler G.S. (1993). General concepts for PCR primer design. PCR Methods Appl..

[10] Dorsett Y., Tuschl T. (2004). siRNAs: Applications in functional genomics and potential as therapeutics. Nat. Rev. Drug Discov..

[11] Lee H.-J. (2014). Additional stories of microRNAs. Exp. Biol. Med..

[12] Hu G., Drescher K.M., Chen X.-M. (2012). Exosomal miRNAs: Biological Properties and Therapeutic Potential. Front. Genet..

[13] Lee Y.S., Pressman S., Andress A.P., Kim K., White J.L., Cassidy J.J., Li X., Lubell K., Lim D.H., Cho I.S. (2009). Silencing by small RNAs is linked to endosomal trafficking. Nat. Cell Biol..

[14] Gibbings D.J., Ciaudo C., Erhardt M., Voinnet O. (2009). Multivesicular bodies associate with components of miRNA effector complexes and modulate miRNA activity. Nat. Cell. Biol..

[15] Pathirana R.D., Kaparakis-Liaskos M. (2016). Bacterial membrane vesicles: Biogenesis, immune regulation and pathogenesis. Cell Microbiol..

[16] Raposo G., Stoorvogel W. (2013). Extracellular vesicles: Exosomes, microvesicles, and friends. J. Cell Biol..

[17] Tricarico C., Clancy J., D’Souza-Schorey C. (2017). Biology and biogenesis of shed microvesicles. Small GTPases.

[18] Panfoli I., Santucci L., Bruschi M., Petretto A., Calzia D., Ramenghi L.A., Ghiggeri G., Candiano G. (2018). Microvesicles as promising biological tools for diagnosis and therapy. Expert Rev. Proteomics.

[19] Zempleni J., Baier S.R., Howard K.M., Cui J. (2015). Gene regulation by dietary microRNAs. Can. J. Physiol. Pharmacol..

[20] Hua C., Zhao J.-H., Guo H.-S. (2018). Trans-Kingdom RNA Silencing in Plant-Fungal Pathogen Interactions. Mol. Plant.

[21] Weiberg A., Wang M., Lin F.-M., Zhao H., Zhang Z., Kaloshian I., Huang H.-D., Jin H. (2013). Fungal small RNAs suppress plant immunity by hijacking host RNA interference pathways. Science.

[22] Arcà B., Colantoni A., Fiorillo C., Severini F., Benes V., Di Luca M., Calogero R.A., Lombardo F. (2019). MicroRNAs from saliva of anopheline mosquitoes mimic human endogenous miRNAs and may contribute to vector-host-pathogen interactions. Sci. Rep..

[23] Bryniarski K., Ptak W., Martin E., Nazimek K., Szczepanik M., Sanak M., Askenase P.W. (2015). Free Extracellular miRNA Functionally Targets Cells by Transfecting Exosomes from Their Companion Cells. PLoS ONE.

[24] Stremersch S., Brans T., Braeckmans K., De Smedt S., Raemdonck K. (2018). Nucleic acid loading and fluorescent labeling of isolated extracellular vesicles requires adequate purification. Int. J. Pharm..

[25] Huang X., Yuan T., Tschannen M., Sun Z., Jacob H., Du M., Liang M., Dittmar R.L., Liu Y., Liang M. (2013). Characterization of human plasma-derived exosomal RNAs by deep sequencing. BMC Genom..

[26] Quek C., Bellingham S.A., Jung C.-H., Scicluna B.J., Shambrook M.C., Sharples R.A., Cheng L., Hill A.F. (2017). Defining the purity of exosomes required for diagnostic profiling of small RNA suitable for biomarker discovery. RNA Biol..

[27] Kourembanas S. (2015). Exosomes: Vehicles of intercellular signaling, biomarkers, and vectors of cell therapy. Annu. Rev. Physiol..

[28] Santangelo L., Giurato G., Cicchini C., Montaldo C., Mancone C., Tarallo R., Battistelli C., Alonzi T., Weisz A., Tripodi M. (2016). The RNA-Binding Protein SYNCRIP Is a Component of the Hepatocyte Exosomal Machinery Controlling MicroRNA Sorting. Cell Rep..

[29] Teng Y., Ren Y., Hu X., Mu J., Samykutty A., Zhuang X., Deng Z., Kumar A., Zhang L., Merchant M.L. (2017). MVP-mediated exosomal sorting of miR-193a promotes colon cancer progression. Nat. Commun..

[30] Cha D.J., Franklin J.L., Dou Y., Liu Q., Higginbotham J.N., Demory Beckler M., Weaver A.M., Vickers K., Prasad N., Levy S. (2015). KRAS-dependent sorting of miRNA to exosomes. Elife.

[31] Villarroya-Beltri C., Gutiérrez-Vázquez C., Sánchez-Cabo F., Pérez-Hernández D., Vázquez J., Martin-Cofreces N., Martinez-Herrera D.J., Pascual-Montano A., Mittelbrunn M., Sánchez-Madrid F. (2013). Sumoylated hnRNPA2B1 controls the sorting of miRNAs into exosomes through binding to specific motifs. Nat. Commun..

[32] Koppers-Lalic D., Hackenberg M., Bijnsdorp I.V., van Eijndhoven M.A.J., Sadek P., Sie D., Zini N., Middeldorp J.M., Ylstra B., de Menezes R.X. (2014). Nontemplated nucleotide additions distinguish the small RNA composition in cells from exosomes. Cell Rep..

[33] Squadrito M.L., Baer C., Burdet F., Maderna C., Gilfillan G.D., Lyle R., Ibberson M., De Palma M. (2014). Endogenous RNAs modulate microRNA sorting to exosomes and transfer to acceptor cells. Cell Rep..

[34] Kang S.-M., Choi J.-W., Hong S.-H., Lee H.-J. (2013). Up-Regulation of microRNA* Strands by Their Target Transcripts. Int. J. Mol. Sci..

[35] Schwechheimer C., Kuehn M.J. (2015). Outer-membrane vesicles from Gram-negative bacteria: Biogenesis and functions. Nat. Rev. Microbiol..

[36] Kim J.H., Lee J., Park J., Gho Y.S. (2015). Gram-negative and Gram-positive bacterial extracellular vesicles. Semin. Cell Dev. Biol..

[37] O’Donoghue E.J., Krachler A.M. (2016). Mechanisms of outer membrane vesicle entry into host cells. Cell Microbiol..

[38] Mulcahy L.A., Pink R.C., Carter D.R.F. (2014). Routes and mechanisms of extracellular vesicle uptake. J. Extracell. Vesicles.

[39] Valadi H., Ekström K., Bossios A., Sjöstrand M., Lee J.J., Lötvall J.O. (2007). Exosome-mediated transfer of mRNAs and microRNAs is a novel mechanism of genetic exchange between cells. Nat. Cell Biol..

[40] Liu S., da Cunha A.P., Rezende R.M., Cialic R., Wei Z., Bry L., Comstock L.E., Gandhi R., Weiner H.L. (2016). The Host Shapes the Gut Microbiota via Fecal MicroRNA. Cell Host Microbe.

[41] Choi J.-W., Kim S.C., Hong S.-H., Lee H.J. (2017). Secretable Small RNAs via Outer Membrane Vesicles in Periodontal Pathogens. J. Dent. Res..

[42] Koeppen K., Hampton T.H., Jarek M., Scharfe M., Gerber S.A., Mielcarz D.W., Demers E.G., Dolben E.L., Hammond J.H., Hogan D.A. (2016). A Novel Mechanism of Host-Pathogen Interaction through sRNA in Bacterial Outer Membrane Vesicles. PLoS Pathog..

[43] Westermann A.J., Förstner K.U., Amman F., Barquist L., Chao Y., Schulte L.N., Müller L., Reinhardt R., Stadler P.F., Vogel J. (2016). Dual RNA-seq unveils noncoding RNA functions in host-pathogen interactions. Nature.

[44] Furuse Y., Finethy R., Saka H.A., Xet-Mull A.M., Sisk D.M., Smith K.L.J., Lee S., Coers J., Valdivia R.H., Tobin D.M. (2014). Search for microRNAs expressed by intracellular bacterial pathogens in infected mammalian cells. PLoS ONE.

[45] Yeri A., Courtright A., Reiman R., Carlson E., Beecroft T., Janss A., Siniard A., Richholt R., Balak C., Rozowsky J. (2017). Total Extracellular Small RNA Profiles from Plasma, Saliva, and Urine of Healthy Subjects. Sci. Rep..

[46] Casadevall A., Pirofski L.A. (2000). Host-pathogen interactions: Basic concepts of microbial commensalism, colonization, infection, and disease. Infect. Immunity.

[47] Drexler S.K., Foxwell B.M. (2010). The role of toll-like receptors in chronic inflammation. Int. J. Biochem. Cell Biol..

[48] Takeuchi O., Akira S. (2010). Pattern recognition receptors and inflammation. Cell.

[49] Mancuso G., Gambuzza M., Midiri A., Biondo C., Papasergi S., Akira S., Teti G., Beninati C. (2009). Bacterial recognition by TLR7 in the lysosomes of conventional dendritic cells. Nat. Immunol..

[50] Li Y., Shi X. (2013). MicroRNAs in the regulation of TLR and RIG-I pathways. Cell. Mol. Immunol..

[51] Cullen B.R. (2006). Viruses and microRNAs. Nat. Genet..

[52] Grundhoff A., Sullivan C.S. (2011). Virus-encoded microRNAs. Virology.

[53] Entwistle L.J., Wilson M.S. (2017). MicroRNA-mediated regulation of immune responses to intestinal helminth infections. Parasite Immunol..

[54] Buck A.H., Coakley G., Simbari F., McSorley H.J., Quintana J.F., Le Bihan T., Kumar S., Abreu-Goodger C., Lear M., Harcus Y. (2014). Exosomes secreted by nematode parasites transfer small RNAs to mammalian cells and modulate innate immunity. Nat. Commun..

[55] Williams M.R., Stedtfeld R.D., Tiedje J.M., Hashsham S.A. (2017). MicroRNAs-Based Inter-Domain Communication between the Host and Members of the Gut Microbiome. Front. Microbiol..

[56] Yuan C., Burns M.B., Subramanian S., Blekhman R. (2018). Interaction between Host MicroRNAs and the Gut Microbiota in Colorectal Cancer. mSystems.

